# Nanotechnology in Cancer Prevention, Diagnosis, and Treatment

**DOI:** 10.3390/pharmaceutics18070892

**Published:** 2026-07-21

**Authors:** Hyun-Ouk Kim

**Affiliations:** 1 Division of Chemical Engineering and Bioengineering, College of Art, Culture and Engineering, Kangwon National University, Chuncheon-si 24341, Gangwon-do, Republic of Korea; kimhoman@kangwon.ac.kr; 2 Department of Smart Health Science and Technology, Kangwon National University, Chuncheon-si 24341, Gangwon-do, Republic of Korea; 3 Institute of Fermentation of Brewing, Kangwon National University, Chuncheon-si 24341, Gangwon-do, Republic of Korea

## 1. Introduction

Nanotechnology has evolved from a promising idea to a working element in how we detect and treat disease. Over the past two decades, the ability to design particles at the nanometer scale has given researchers a way to carry drugs to places that were once hard to reach, to sharpen the signal in diagnostic tests, and to combine therapy and imaging in a single construct. Cancer has been one of the main proving grounds for this work, largely because tumors present problems that conventional drugs struggle with, including poor selectivity, rapid clearance, and resistance.

This Special Issue of *Pharmaceutics*, entitled “Nanotechnology in Cancer Prevention, Diagnosis, and Treatment,” was assembled to capture how nanoscale systems address the specific demands of oncology. Eleven contributions were gathered from groups working on several continents, spanning detection, prevention, and treatment. This editorial provides a short tour through the study directions represented in each paper.

## 2. Overview of the Contributions

The contributions can be read as a progression from detection to prevention and then on to treatment. On the diagnostic side, Song and colleagues developed a gold nanoparticle-linked immunosorbent assay that detects staphylococcal enterotoxin B with very high sensitivity by measuring gold atom content, showing how nanoparticles can push the detection limit of established assay formats (Contribution 1). Zheng and co-workers reviewed nano- and micromotors for cancer diagnosis and therapy, with attention to the biocompatibility problems that keep these small machines in the laboratory rather than the clinic (Contribution 5).

Prevention is a less common theme in cancer nanomedicine, which makes the work here worth noting. Long and colleagues showed that orally administered M13-loaded lipid nanoparticles can help prevent colitis-associated cancer, connecting gut biology, microbial metabolites, and colon-targeted delivery (Contribution 3). On the treatment side, the contributions span several strategies. Chandrasekar and co-workers used a calreticulin-inducing nanoparticle to overcome resistance to immune checkpoint inhibitors, turning a poorly responsive tumor into one that reacts to therapy (Contribution 2). Kim and colleagues reported charge-complementary polymersomes, described as ChargeSomes, that protect mRNA from degradation and improve its delivery into cells, an approach with clear relevance to both cancer vaccines and broader mRNA therapy (Contribution 4). Zhou and co-workers reviewed second near-infrared window photothermal agents and their use in photothermal therapy, a modality valued for its tumor-killing capacity and limited side effects (Contribution 11).

This Special Issue also gathered works that expand the toolbox, including light-responsive multifunctional nanofibers for targeted delivery (Contribution 6), a focused review of delivery strategies for BCG-unresponsive bladder cancer (Contribution 7), spleen-targeted delivery and its implications for cancer (Contribution 10), and nanotechnological advances built around the natural product Ganoderma lucidum (Contribution 9). A study on fluorescent Rhein-loaded liposomes rounds out the diagnostic and biodistribution side of the collection (Contribution 8).

## 3. Outlook and Future Directions

Reading these eleven contributions together, several gaps stand out as natural targets for future study. Biocompatibility and long-term safety remain a recurring obstacle, named directly in the work on nano- and micromotors but relevant to nearly every platform discussed here. Delivery specificity is a second theme, whether the target is a solid tumor, the spleen, or a premalignant lesion in the colon. A third question, harder to answer, is how well the results from in vitro models and animal studies will hold up in patients.

The direction of travel seems clear. mRNA payloads, immune-activating constructs, and combination approaches that pair targeted delivery with checkpoint modulation are likely to draw the most attention in the coming years, and careful formulation science and honest safety assessment will matter as much as new materials. It is my hope that this Reprint gives readers both a snapshot of where cancer nanomedicine stands and a sense of the questions worth pursuing next.

